# Assessing the mitochondrial DNA diversity of the Chagas disease vector *Triatoma sordida* (Hemiptera: Reduviidae)

**DOI:** 10.1590/0074-02760150429

**Published:** 2016-05

**Authors:** Grasielle Caldas D‘Ávila Pessoa, Tais Nóbrega de Sousa, Ivan Vieira Sonoda, Liléia Diotaiuti

**Affiliations:** 1 Fundação Oswaldo Cruz, Centro de Pesquisas René Rachou, Laboratório de Referência em Triatomíneos e Epidemiologia da Doença de Chagas, Belo Horizonte, MG, Brasil; 2 Fundação Oswaldo Cruz, Centro de Pesquisas René Rachou, Laboratório de Malária, Belo Horizonte, MG, Brasil

**Keywords:** Triatominae, Triatoma sordida, cyt *b* diversity

## Abstract

*Triatoma sordida* is a species that transmits *Trypanosoma cruzi* to humans. In Brazil, *T. sordida* currently deserves special attention because of its wide distribution, tendency to invade domestic environments and vectorial competence. For the planning and execution of control protocols to be effective against Triatominae, they must consider its population structure. In this context, this study aimed to characterise the genetic variability of *T. sordida* populations collected in areas with persistent infestations from Minas Gerais, Brazil. Levels of genetic variation and population structure were determined in peridomestic *T. sordida* by sequencing a polymorphic region of the mitochondrial cytochrome *b* gene. Low nucleotide and haplotype diversity were observed for all 14 sampled areas; π values ranged from 0.002-0.006. Most obtained haplotypes occurred at low frequencies, and some were exclusive to only one of the studied populations. Interpopulation genetic diversity analysis revealed strong genetic structuring. Furthermore, the genetic variability of Brazilian populations is small compared to that of Argentinean and Bolivian specimens. The possible factors related to the reduced genetic variability and strong genetic structuring obtained for studied populations are discussed in this paper.

In Brazil, four species currently deserve special attention in *Trypanosoma cruzi* transmission to humans: *Triatoma brasiliensis*, *Panstrongylus megistus*, *Triatoma pseudomaculata* and *Triatoma sordida* (MS 2015). The Reduviid bug *T. sordida* (Stal, 1859) is endemic in the Cerrado, the main biome of Central Brazil from which dispersion towards the southwest took place, and it is now widely distributed throughout Argentina, Bolivia, Paraguay and Uruguay (Forattini 1980, Carcavallo et al. 1997, Galvão & Gurgel-Gonçalves 2015). A recent study of ecological niche modelling revealed the possibility that *T. sordida* is distributed over an area greater than initially thought, and it may be present in other biomes (e.g., Caatinga and Pantanal) (Gurgel-Gonçalves et al. 2012, Galvão & Gurgel-Gonçalves 2015).

*T. sordida* is considered a ubiquitous species with high ecological potential that can live in various ecotopes and feed from different sources. This insect can withstand large environmental changes that cause its competitors to disappear and can widen its ecotopes to include dry trees and dead trees (Forattini et al. 1974). The epidemiological importance of *T. sordida* is increasing due to its tendency to invade houses, particularly in areas where *Triatoma infestans* has been controlled. This process of domiciliation may merely reflect the invasion of habitats from which *T. infestans* has been eliminated, but it may also be primary, without any relation to the previous eradication of the main vector (Forattini et al. 1984, Noireau et al. 1996).

In artificial environments, given the frequency with which it has been found outside the peridomicile and intradomicile, *T. sordida* is considered a semidomiciliar species (Coura 1993) and is present in some situations at high densities (Diotaiuti & Pinto 1991). *T. sordida* is associated with the reinfestation of dwellings treated with insecticides (Pessoa et al. 2015a). The dispersal pattern of this triatomine is linked to increased infestations of dwellings closest to sylvatic environments and suggests that recolonisation flows to artificial environments from natural ecotopes (Forattini et al. 1974, Diotaiuti et al. 1998).

Considering the wide distribution of *T. sordida*, its tendency to invade domestic environments, and its vectorial competence in the laboratory, we consider it a triatomine that has potential epidemiological importance (Forattini et al. 1974, Diotaiuti 1995, Rojas de Arias et al. 2012). In 2008, in Ibipitanga, Brazil, oral transmissions of Chagas disease occurred from the ingestion of sugarcane juice prepared in an abandoned sugarcane mill where specimens of *T. sordida* contaminated with *T. cruzi* were captured (Dias et al. 2008). These findings emphasise the necessity to evaluate the importance of vectors such as *T. sordida* in maintaining the endemicity of this disease. Thus, it is important to investigate aspects regarding the planning and execution of vector control initiatives including the assessment of levels of genetic variation, population structure and gene flow among insect populations (Costa et al. 1997, Noireau et al. 1999, Borges et al. 2000a, b, Marcilla et al. 2002, Barbosa et al. 2003, 2006, Almeida et al. 2008, Kopp et al. 2009, Cavassin et al. 2014, González-Brítez et al. 2014, Panzera et al. 2015).

Therefore, this study aimed to characterise the genetic variability of *T. sordida* populations collected in persistently infested areas of Minas Gerais state, Brazil, using the mitochondrial (mt) cytochrome *b* gene (cyt *b*). To our knowledge, this is the first study to characterise the genetic diversity of insects from areas with reports of persistent triatomine reinfestations despite the chemical control activities existence in accordance with the Brazilian Ministry of Health.

## MATERIALS AND METHODS

**Fig. 1:**
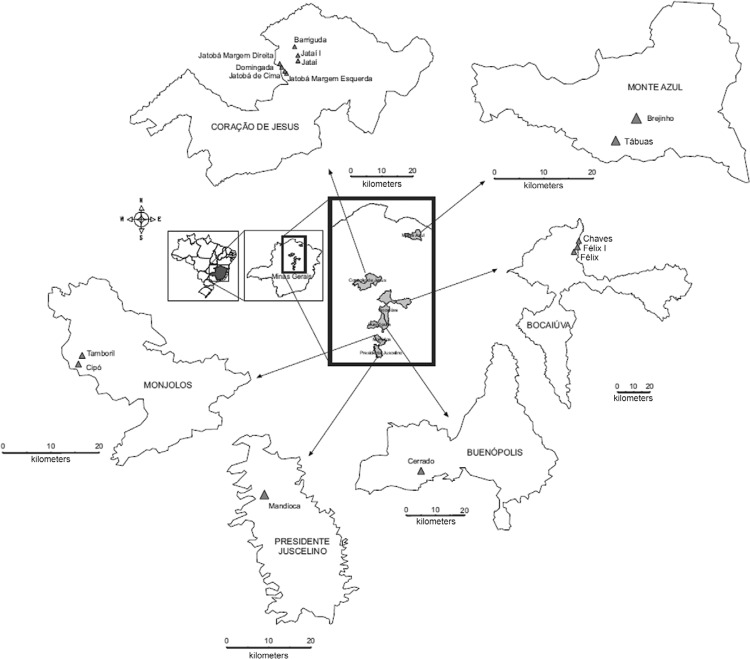
: map of Minas Gerais, Brazil, showing the study collection areas for *Triatoma sordida* populations.

*Insects’ origins* - The studied populations were manually collected in 2007 with the assistance of technicians from Gerência Regional de Saúde de Montes Claros and Sete Lagoas, Minas Gerais (MG), Brazil, without using a dislodging agent. The insects came from peridomiciles in the central (Monjolos - 18º 19’ 30” S 44º 07’ 08” O; Presidente Juscelino - 18º 38’ 13” S 44º 03’ 28” O; Buenópolis - 17º 52’ 22” S 44º 10’ 48” O) and northern (Monte Azul - 15º 09’ 18” S 42º 52’ 30” O; Coração de Jesus - 16º 41’ 06” S 44º 21’ 54” O; and Bocaiúva - 17º 06’ 28” S 43º 48’ 54” O) areas of MG state, Brazil (Fig. 1). In these areas a Chagas Disease Control Programme was undertaken, and applied continuously and systematically over the last 30 years through applications of residual insecticides. Adults and nymphs of the parental generation were used to perform the experiments.

*DNA extraction, amplification and sequencing* - Genomic DNA extractions from legs (two per specimen) were performed using the protocol described by Balbino et al. (2006). The samples were subjected to polymerase chain reaction (PCR) using primers targeting the cyt *b* gene: CYT BF-5` GGACAAATATCATGAGGAGCAACAG 3` and CYT BR-5` ATTACTCCTCCTAGCTTATTAGGAATTG 3` (Lyman et al. 1999). Briefly, all PCR reactions were performed in 20 μL volumes containing 1 pmol of each primer: 1.5 U Taq DNA Polymerase (Invitrogen, La Jolla, CA), 3 mM MgCl_2_, 0.2 mM of each dNTP and ~30 ng DNA. Amplifications were carried out in an Eppendorf Mastercycler Gradient (Eppendorf, Germany) using the following reaction conditions: 95ºC for 5 min, 30 cycles at 95ºC for 45 s, annealing at 50ºC for 45 s, 72ºC for 1 min, and a final extension step at 72ºC for 10 min. The amplified PCR products were purified using a GFX-96 PCR kit (Amersham Biosciences, Little Chalfont, UK) and directly sequenced with specific primers (CYT BF and CYT BR) using a DYEnamic^TM^ ET dye terminator kit (Amersham Biosciences, Little Chalfont, UK). The products were analysed on a MegaBace 500 automated DNA sequencer (Amersham). The isolate sequences were sequenced at least twice for each strand from independent PCR amplifications.

*Sequence analysis* - Sequence alignment was performed using the MUSCLE multiple alignment program (Edgar 2004). The number of segregating sites and haplotypes, as well estimates of nucleotide diversity (π, average number of substitutions between any two sequences, assuming that the sample is random) and their standard deviations were calculated using DnaSP 5.1 software (Librado & Rozas 2009). Between-population differentiations were measured using the pairwise fixation index (F_ST_) (Wright 1951) with Arlequin 3.5 software (Excoffier & Lischer 2010). The correlation between pairwise population genetic distances (F_ST_) and geographical distances was estimated by nonparametric Spearman’s rank correlation. Phylogenetic trees were reconstructed by the maximum likelihood method in PhyML 3.0 (Guindon et al. 2010) using the Hasegawa-Kishino-Yano model with gamma distributed rate variation among sites (Hasegawa et al. 1985). jModeltest was used to assess the best fit model of nucleotide substitution (Posada 2008). The reliability of clustering patterns in trees was assessed by 1,000 bootstrap replicates (Felsenstein 1985). We used the cyt *b* sequences of *P. megistus* (GenBank accession number: AF045722.1) and *Rhodnius prolixus* (EF011726.1) as outgroups. A sequence of Bolivian isolate (AF045730.1) was used as a reference in this study (Monteiro et al. 1999). The complete description of the sequences analysed here is shown in Supplementary Table. This study was approved by the Animal Ethics Committee of Fundação Oswaldo Cruz (number 29/14-1).

## RESULTS

*Nucleotide and haplotype diversity of cyt b among Brazilian isolates* - We sequenced a fragment consisting of 233 bp of the cyt *b* gene from 126 isolates of *T. sordida* originating from 14 different areas of MG state in southwestern Brazil. The sequenced region (from nt 86-318) corresponds to the most polymorphic portion of the gene. 13 polymorphic sites (six synonymous substitutions and seven nonsynonymous substitutions) were identified, with an overall nucleotide diversity of 0.004 (Table I). For all 14 sampled areas of MG, low nucleotide diversity was estimated with π values ranging from 0.002-0.006. We also analysed five sequences of cyt *b* available in GenBank of isolates from Bolivia. 28 polymorphic sites were identified in the same 233 bp. We observed extensive variability with an overall nucleotide diversity of 0.053 in the Bolivian samples.

**TABLE I:** Description of cytochrome *b* polymorphisms identified in *Triatoma sordida* isolates from Minas Gerais state, Brazil

	Nucleotide^a^	93	97	111	114	118	161	246	255	306	308	310	312	318
Haplotype	*T. sordida*	ACA ^c^ (T/T)^d^	TTT (F/I)	CAC (H/H)	TTC (F/L)	CTC (L/I)	TTC (F/S)	AAG (K/N)	ATA (M/M)	CCC (P/P)	CGC (R/H)	ATC (I/F)	ATC (I/I)	GGA (G/G)
	1 (74)^b^	G	-	-	-	-	-	-	G	T	-	-	-	-
	2 (10)	G	-	-	-	-	-	-	G	T	-	-	-	G
	3 (1)	G	-	-	A	-	-	-	G	T	A	-	-	-
	4 (4)	G	-	-	-	-	-	-	G	T	A	-	-	-
	5 (9)	-	-	-	-	-	-	-	G	T	-	-	-	-
	6 (1)	-	-	-	-	-	C	-	G	T	-	-	-	-
	7 (1)	-	-	-	-	-	-	T	G	T	-	-	-	-
	8 (13)	G	-	-	-	-	-	-	G	T	-	T	-	-
	9 (7)	G	-	-	-	-	-	-	G	-	-	-	-	-
	10 (1)	G	A	-	-	-	-	-	G	T	-	-	-	G
	11 (1)	G	-	-	-	A	-	-	G	T	-	-	-	-
	12 (1)	G	-	-	-	-	-	-	G	T	-	-	T	-
	13 (1)	G	-	T	-	-	-	-	G	T	-	-	-	-
	14 (1)	G	A	T	-	-	-	-	G	T	-	-	-	-
	15 (1)	G	-	-	-	-	-	-	-	T	-	-	-	-

*a*: nucleotide numbers according to sequence of *T. sordida* isolate used as reference in this study (GenBank accession number AF045730.1); *b*: the number of Brazilian isolates characterised by the haplotype is indicated in parenthesis. A description of the haplotypes is available in Supplementary Table; *c*: the codon position and the nucleotide substitution are shown in bold underlined text. Dots indicate identical nucleotides of the reference isolate; *d*: the first amino acid corresponds to the sequence of the reference isolate, while the second is the polymorphic amino acid obtained from the Brazilian isolates.

**Fig. 2:**
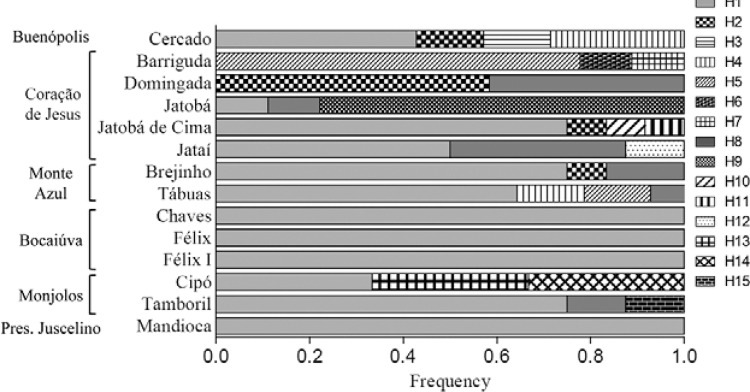
: haplotype frequencies of cytochrome *b* in Minas Gerais state, Brazil. The scale bar shows the frequency of each haplotype per area indicated in the horizontal boxes.

The polymorphisms obtained were arranged in 15 haplotypes (Table I, Fig. 2), corresponding to a mean haplotype diversity of 0.633. Among these haplotypes, only five (H1, H2, H5, H8 and H9) had frequencies above five percent. A single haplotype (H1) was predominant in 60% of the isolates, which were distributed across all but two of the sampled areas (Fig. 2). With few exceptions, H1 was the most frequent haplotype in the areas where it occurred. Different unique patterns of haplotypic variation with other predominant haplotypes were observed in Jatobá, Domingada and Barriguda (municipality of Coração de Jesus). The cyt *b* haplotype sequences obtained here were deposited in GenBank with accession numbers KR822185, KR822186, KR822187, KR822188, KR822189, KR822190, KR822191, KR822192, KR822193, KR822194, KR822195, KR822196, KR822197, KR822198 and KR822199.

*Interpopulation genetic diversity* - Comparative analyses of genetic variability between populations of *T. sordida* from the 14 areas in MG state showed that three populations (Jatobá, Barriguda and Domingada) are distinctly different from all other studied populations (Table II). The F_ST_ values of these populations ranged from 0.29-0.80. On the other hand, for the majority of populations, we observed overall low genetic differentiation and F_ST_ values were not significant (p > 0.05). Moreover, the F_ST_ values did not correlate with the geographic distance between populations (Spearman correlation coefficient = -0.160, p = 0.129).

**TABLE II:** Intra- and interpopulation variability of *Triatoma sordida* from Brazil

Geographical location (N)		S	Π (SD)					F_ST_								
				Cerrado	Barriguda	Domingada	Jatobá	Jatobá de Cima	Jataí	Brejinho	Tábuas	Chaves	Félix	Félix I	Cipó	Tamboril
Buenópolis	Cercado (7)	3	0,005 (0,001)	-	-	-	-	-	-	-	-	-	-	-	-	-
Coração de Jesus	Barriguda (9)	2	0,002 (0,001)	0,40	-	-	-	-	-	-	-	-	-	-	-	-
	Domingada (12)	2	0,005 (0,001)	0,29	0,52	-	-	-	-	-	-	-	-	-	-	-
	Jatobá (9)	2	0,003 (0,001)	0,37	0,58	0,50	-	-	-	-	-	-	-	-	-	
	Jatobá de Cima (12)	3	0,003 (0,001)	0,07	0,56	0,48	0,52	-	-	-	-	-	-	-	-	-
	Jataí (8)	2	0,003 (0,001)	0,05	0,46	0,29	0,40	0,10	-	-	-	-	-	-	-	-
Monte Azul	Brejinho (12)	2	0,002 (0,001)	0,09	0,57	0,45	0,52	-0,04	0,02	-	-	-	-	-	-	-
	Tábuas (14)	3	0,003 (0,001)	-0,01	0,43	0,43	0,45	0,00	0,04	-0,01	-	-	-	-	-	-
Bocaiúva	Chaves (10)	0	-	0,36	0,80	0,71	0,78	0,07	0,36	0,10	0,14	-	-	-	-	-
	Félix (8)	0	-	0,32	0,78	0,69	0,75	0,04	0,32	0,07	0,12	0,00	-	-	-	-
	Félix 1 (7)	0	-	0,29	0,77	0,68	0,74	0,03	0,30	0,05	0,10	0,00	0,00	-	-	-
Monjolos	Cipó (3)	2	0,006 (0,002)	-0,03	0,40	0,33	0,38	0,15	0,04	0,17	0,08	0,62	0,56	0,52	-	-
	Tamboril (8)	2	0,002 (0,001)	0,07	0,56	0,47	0,51	-0,05	0,01	-0,08	-0,03	0,11	0,07	0,05	0,12	-
Presidente Juscelino	Mandioca (7)	0	-	0,29	0,77	0,68	0,74	0,03	0,30	0,05	0,10	0,00	0,00	0,00	0,52	0,05

N: number of isolates sequenced; S: number of segregating (polymorphic/variable) sites; π: observed average pairwise nucleotide diversity; SD: standard deviation; F_ST_: fixation index, a measure of genetic differentiation between populations (underlined values indicate that p < 0.05).

**Fig. 3:**
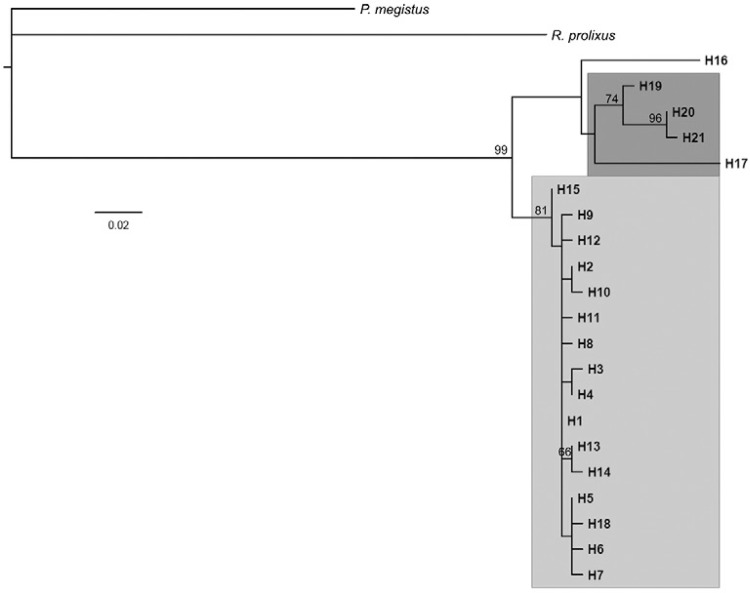
: best maximum likelihood tree reconstructed. The numbers above the branches represent clade support higher than 50. The description of haplotypes is shown in Supplementary Table. The cytochrome *b* sequences of *Panstrongylus megistus* and *Rhodnius prolixus* were used as an outgroup. Clades were highlighted according to the geographic origin of haplotypes: dark grey = Bolivia; light grey = Brazil.

*Phylogenetic analysis* - The phylogenetic relationships among *T. sordida* isolates from different areas including Brazil, Argentina and Bolivia were inferred from the cyt *b* sequences (Fig. 3). The Brazilian isolates felt into a group of isolates separated from those from the other countries with high support (bootstrap value of 81%). Only one sample from Bolivia (haplotype 18) clustered with the same group of Brazilian isolates. The other Bolivian isolates, represented by haplotypes 19, 20 and 21, were placed in a separate group with high support.

## DISCUSSION

There is no doubt that chemical control of triatomine populations was successful in most Southern Cone countries, resulting in Uruguay, Chile and Brazil being certified as free of vector transmission by *T. infestans* (Dias 2006). Parallel *T. infestans* elimination effort in Brazil, was also reduced the density of other triatomine species (Dias et al. 2002). However, reinfestation by native triatomines, with a high capacity for invasion/colonisation of domestic units persists in Brazil in substantially different epidemiological settings, requiring individual evaluations of the various scenarios. Persistent reinfestations that particularly stand out include those by *T. brasiliensis* in semiarid areas, by *P. megistus* in areas associated with residual forests and by *T. sordida* in the Cerrado (Pessoa et al. 2015b).

In this sense, despite the importance of knowing the genetic structure and dynamics of triatomine infestation/reinfestation for the design of chemical control activities, there are few studies in the literature. Studies with Brazilian populations of *P. megistus* using isoenzymes (Kopp et al. 2009), Random Amplified Polymorphic DNA (RAPD) (Barbosa et al. 2003, 2006) and ribosomal intergenic sequences (ITS1 and ITS2) (Cavassin et al. 2014) revealed populations with strong population structure and reduced genetic diversity that was directly related to the geographic distance between the studied areas. This same pattern was observed in Brazilian populations of *T. brasiliensis* using isoenzymes (Costa et al. 1997), RAPD (Borges et al. 2000a, b) and the cyt *b* gene (Monteiro et al. 2004, Almeida et al. 2008).

To date, there has been only one study that investigated the genetic diversity of natural Brazilian populations of *T. sordida* (Monteiro et al. 2009). Monteiro et al. (2009) determined the genetic variation levels and the population structure for 181 specimens of *T. sordida* collected from four municipalities of MG state (Espinosa, Mamonas, Januária and Corinto) by analysing 28 allozyme loci. None of these loci presented fixed differences between any pair of populations, and only two revealed polymorphisms, accounting for extremely low levels of heterozygosity (He = 0.027). Regardless of the low levels of polymorphism obtained, the results indicated the existence of genetic structure among the populations analysed (F_ST_ = 0.214). In turn, the studies using isoenzymes (Noireau et al. 1999 **),** RAPD (González-Brítez et al. 2014) showed similar results for *T. sordida* populations in Bolivia and Paraguay, respectively.

Corroborating Monteiro et al. (2009), we report that the genetic structure of natural Brazilian populations of *T. sordida* has low levels of genetic variability. Overall, the sequencing of a polymorphic fragment of the cyt *b* gene of *T. sordida* from 14 areas of MG, Brazil, showed low genetic diversity in the 126 isolates analysed. The nucleotide diversity of isolates from Bolivia was approximately 13 times greater than that of isolates from Brazil. The analysis of haplotypic data confirmed these results. A predominant haplotype (H1) was obtained in 60% of samples. Most of the 15 identified haplotypes were observed at low frequency, and some were exclusive to only one of the study populations. It is noteworthy that we obtained a very different haplotype profile for the three populations from Coração de Jesus municipality (Barriguda, Domingada and Jatobá). The interpopulation genetic diversity analysis also revealed strong genetic structuring - particularly in populations from Coração de Jesus - compared with other study populations of this insect vector (F_ST_ > 0.3).

The low genetic diversity and strong genetic structuring of the population samples from MG may be related to different factors, alone or in combination, as follows: (i) possible geographical isolation due to an obstacle to triatomine flow between neighbouring localities (Noireau et al. 1998, González-Brítez 2013, González-Brítez et al. 2014); (ii) focal distribution of insects in small colonies usually comprised of a few individuals, thereby limiting gene flow between them; (iii) the low dispersal capacity of *T. sordida* (Monteiro et al. 2009); and (iv) the long Triatominae life cycle, which ensures that contributions from young adults able to reproduce (and consequently exchange genetic material) occurs over long intervals that differ among triatominic species (Forattini et al. 1974). Moreover, considering that this area has suffered continuous pressure from insecticides used since the 1950s to triatomines control (Silveira 1994, Mendes 2008, Pessoa 2012), it could be expected that the studied populations would show a low genetic variability. There is evidence that genetic diversity is reduced in areas treated with insecticides compared with untreated areas due to bottleneck events (Pérez de Rosas et al. 2007, 2008). In this context, the study area of this work overlaps with the dengue and leishmaniasis endemics. These programmes execute their vector chemical control activities simultaneously and independently. In addition, the utilisation of agricultural and domestic insecticides exacerbates the chemical pressure on triatomine populations of the area, which can contribute to indiscriminate and unwanted increases in insecticide resistance (Pessoa 2012). Pessoa et al. (2015a) - for the same populations studied in this work - revealed the largest resistance ratios ever identified for populations of *T. sordida* (RR_50_ 2.5-7.2). Of the 14 studied populations, all populations presented equal or higher slopes compared to the Susceptibility Reference Lineage (SRL), suggesting low genetic variation when compared to SRL. Moreover, different deltamethrin susceptibility profiles were identified in populations from distinct locations that, nevertheless, belong to the same municipalities (ex. Localities of Coração de Jesus), reinforcing the complexity of the resistance phenotype, not only at the macrogeographical level, but at the microgeographical level. It should be noted that the insecticide used in the field does not appear to have homogeneous effects over different populations; consequently, it applies different selection pressures to different populations, which is a reflection of the genetic variability among those same populations. The complexity of the peridomicile itself cannot be neglected, either. The large variety of ecotopes that exist in peridomiciles makes spraying these ecotopes an exhaustive job. Separating all the material accumulated there for spraying is operationally impossible for the responsible health agent. Consequently, *T. sordida* (eggs, nymphs and adults) remain even after the application of the insecticide, hidden deep in piles of firewood, under barn roofs and in a variety of other nearly inaccessible places, staying free from contact with the active chemical and/or in contact only with sublethal doses, which favours their multiplication in these ecotopes (Diotaiuti et al. 1998). In this case, triatomine populations may be formed from the surviving specimens and could found a new colony - with reduced genetic variability.

For populations with greater relative haplotype diversity, one should consider possible flows of insects from wild to domestic environments that occur in response to environmental changes caused by human action in the study area. In agricultural areas and livestock operations, substantial modifications to the natural environment have led to the displacement or disappearance of ref- uges and natural food sources of *T. sordid*a. As a result, the insect seeks artificial alternative environments in which it can survive. It appears as if changes in vegetation coverage, at least to some extent, cause dispersion of *T. sordida*. Wide infestation in households closer to wild environments suggests a triatomine (nymph to adult) recolonisation flow to artificial environments from natural ecotopes (Forattini et al. 1971), thus contributing to the observed persistent reinfestations in the area.

The phylogenetic analysis performed in this study showed that *T. sordida* specimens from Argentina and Bolivia are grouped into a separate cluster than the Brazilian populations. Although only one Argentinean sample and five Bolivian samples were compared, there was a closer relationship between the Argentinian specimen and most Bolivian samples of *T. sordida*. However, one Bolivian isolate was grouped with the Brazilian samples. Cytogenetic studies combined with isoenzymes using Argentinean and Brazilian populations of *T. sordida* corroborate the results of the present study and also showed high levels of genetic differentiation (Panzera et al. 1997). In addition, Justi et al. (2014) compared the genetic variability among eight specimens of *T. sordida* from Brazil, Bolivia and Argentina using different mt markers (16S, cyt oxidase I, cyt oxidase II and cyt *b*) and two nuclear markers (18S and 28S), showing that the *T. sordida* from group two were restricted to Chaco, while those from group one were restricted to Bolivia and Brazil (Forattini 1980, Noireau et al. 1996).

## SUPPLEMENTARY TABLE

**Table:** Description of cytochrome *b* haplotypes of *Triatoma sordida* isolates

Haplotype identification	Number of isolates	Isolates description (Accession number)^b^	Geographical origin	

			Country	Municipality^c^
H1^a^	77	KR822185; KC249289; KC249292; KC249294	Brazil	Buenópolis/Cerrado (3), Coração de Jesus/Jatobá (1)/Jatobá de Cima (9)/Jataí (4), Bocaiúva/Chaves (10)/Félix (8)/Félix I (7), Monte Azul/Brejinho (9)/Tábuas (9), Monjolos/Cipó (1)/Tamboril (6), Presidente Juscelino/Mandioca (7), Others regions
H2	11	KR822186; KC608980		Buenópolis/Cerrado (1), Coração de Jesus/Domingada (7)/Jatobá de Cima (1), Monte Azul/Brejinho (1), Others regions
H3	1	KR822187		Buenópolis/Cerrado
H4	4	KR822188		Buenópolis/Cerrado (2), Monte Azul/Tábuas (2)
H5	9	KR822189		Coração de Jesus/Barriguda (7), Monte Azul/Tábuas (2)
H6	1	KR822190		Coração de Jesus/Barriguda
H7	1	KR822191		Coração de Jesus/Barriguda
H8	13	KR822192		Coração de Jesus/Domingada (5)/Jatobá (1)/Jataí (3), Monte Azul/Brejinho (2)/Tábuas (1), Monjolos/Tamboril (1)
H9	7	KR822193		Coração de Jesus/Jatobá
H10	1	KR822194		Coração de Jesus/Jatobá de Cima
H11	1	KR822195		Coração de Jesus/Jatobá de Cima
H12	1	KR822196		Coração de Jesus/Jataí
H13	1	KR822197		Monjolos/Cipó
H14	1	KR822198		Monjolos/Cipó
H15	1	KR822199		Monjolos/Tamboril
H16	1	KC249295	Argentina	
H17	1	KC249293	Bolivia	
H18	1	KC249291	Bolivia	
H19	1	HQ333243	Bolivia	
H20	1	HQ333242	Bolivia	
H21	1	AF045730	Bolivia	

*a*: the haplotypes were reconstructed from a sequence of 233 bp (from nt 86-318) analysed from the cyt *b* gene and correspond to the haplotypes used to infer the phylogenetic relationships among *T. sordida* isolates; *b*: GenBank accession numbers of the haplotype sequences of cyt *b* from Brazilian isolates and isolate sequences available from GenBank from other regions used in our analysis; *c*: the number of Brazilian isolates characterised by the haplotype is indicated in parentheses.
